# Organoids: opportunities and challenges of cancer therapy

**DOI:** 10.3389/fcell.2023.1232528

**Published:** 2023-07-27

**Authors:** Xianjie Jiang, Linda Oyang, Qiu Peng, Qiang Liu, Xuemeng Xu, Nayiyuan Wu, Shiming Tan, Wenjuan Yang, Yaqian Han, Jinguan Lin, Longzheng Xia, Mingjing Peng, Yanyan Tang, Xia Luo, Min Su, Yingrui Shi, Yujuan Zhou, Qianjin Liao

**Affiliations:** ^1^ Hunan Key Laboratory of Cancer Metabolism, Hunan Cancer Hospital, Affiliated Cancer Hospital of Xiangya School of Medicine, Central South University, Changsha, Hunan, China; ^2^ Public Service Platform of Tumor Organoids Technology, Changsha, Hunan, China; ^3^ Hengyang Medical School, University of South China, Hengyang, Hunan, China

**Keywords:** organoids, organoids development, establishing, application, cancer therapy

## Abstract

Organoids are a class of multicellular structures with the capability of self-organizing and the characteristic of original tissues, they are generated from stem cells in 3D culture *in vitro*. Organoids can mimic the occurrence and progression of original tissues and widely used in disease models in recent years. The ability of tumor organoids to retain characteristic of original tumors make them unique for tumorigenesis and cancer therapy. However, the history of organoid development and the application of organoid technology in cancer therapy are not well understood. In this paper, we reviewed the history of organoids development, the culture methods of tumor organoids establishing and the applications of organoids in cancer research for better understanding the process of tumor development and providing better strategies for cancer therapy. The standardization of organoids cultivation facilitated the large-scale production of tumor organoids. Moreover, it was found that combination of tumor organoids and other cells such as immune cells, fibroblasts and nervous cells would better mimic the microenvironment of tumor progression. This might be important developing directions for tumor organoids in the future.

## Introduction

Cancer is now a major cause of death worldwide and an important barrier for extending life expectancy (Siegel et al., 2023). According to World Health Organization’s data about cancer statistics for 183 countries in 2019, the mortality caused by cancer was high among those who died before the age of 70, and in some countries it even ranked first (Sung et al., 2021). Studying tumor development process, developing tumor early diagnosis technology and improving tumor treatment efficiency are directions of current cancer research. Among them, the establishment of tumor model is the most critical factor (Balani et al., 2017; Drost and Clevers, 2018). Traditional 2 dimensional (2D) culture model of tumor cells had the characteristics of easy to handle and rapid to culture, which was widely used in tumor research and anti-tumor drug screening. However, there were two deficiencies in the 2D cells culture model, one was poor tumor heterogeneity, the other was new mutation produced during the culture process, which were the main reasons for poor efficiency of antitumor drugs in clinical trials (Kretzschmar, 2021). PDX (patient derived xenograft) model can effectively replicate tumor growth and preserve the heterogeneity of tumors in the tumor microenvironment, making it an ideal model for studying tumors and screening anti-tumor drugs. However, due to the low success rate of PDX model established, long time spent and huge costed, it was difficult to apply them on large scale applications (Ben-David et al., 2017; Byrne et al., 2017). The emergence of organoid model can greatly compensate for the shortcomings of 2D cell model and PDX model, and it has great potential in disease model constructing, anti-tumor drug screening and organ transplantation.

Organoids are multicellular mass formed by 3D (3 dimensional) culture of stem cells (including embryonic stem cells, induced pluripotent stem cells or adult tissue stem cells) *in vitro*, which have the ability to self-organize and self-renew. Furthermore, they can mimic the structure and function of the original tissues (Camp et al., 2017). Compared to traditional 2D cells model (e.g., cell lines, original cells), organoid model contains multiple cell compositions and 3D culture environments which better reflect the structural and functional characteristics of the original tissue so that they can maintain genetic and phenotypic stability during long-term expansion (Kaluthantrige Don and Huch, 2021). It largely overcomes the deficiency of stable genetic amplification arising from the process of 2D culture of cells. At the same time, the interaction and communication between cells and the pericellular matrix are preserved, which greatly reflects the growth of tissues in the body. Moreover, the organoid model is superior to PDX model because it is cultivated *in vitro*, it means that organoid model will be more cost-effective and rapidly in cultivation. The organoid technology had also been selected by several top journals as a major breakthrough in science (Rossi et al., 2018).

Currently, organoids had involved in many aspects of medicine and played an important role in basic and clinical medical research, especially in the study of tumorigenesis, tumor heterogeneity and tumor precision therapy. However, there were some shortcomings in the applications of organoids in tumors, such as the current inconsistency of tumor culture methods and poor reproducibility were the major drawbacks of organoids. In addition, the lack of fibroblasts and immune cells in the organoids lead to the inability of the organoids to truly reflect tumor microenvironment on tumor cells. This paper reviewed the history of organoids development, the establishment methods of tumor organoids, the applications of organoid technology in tumor treatment and the shortcomings of current tumor organoids. Moreover, we summarized the methods to compensate the deficiencies and discussed the future development directions of tumor organoids.

### History of organoids development

**FIGURE 1:**
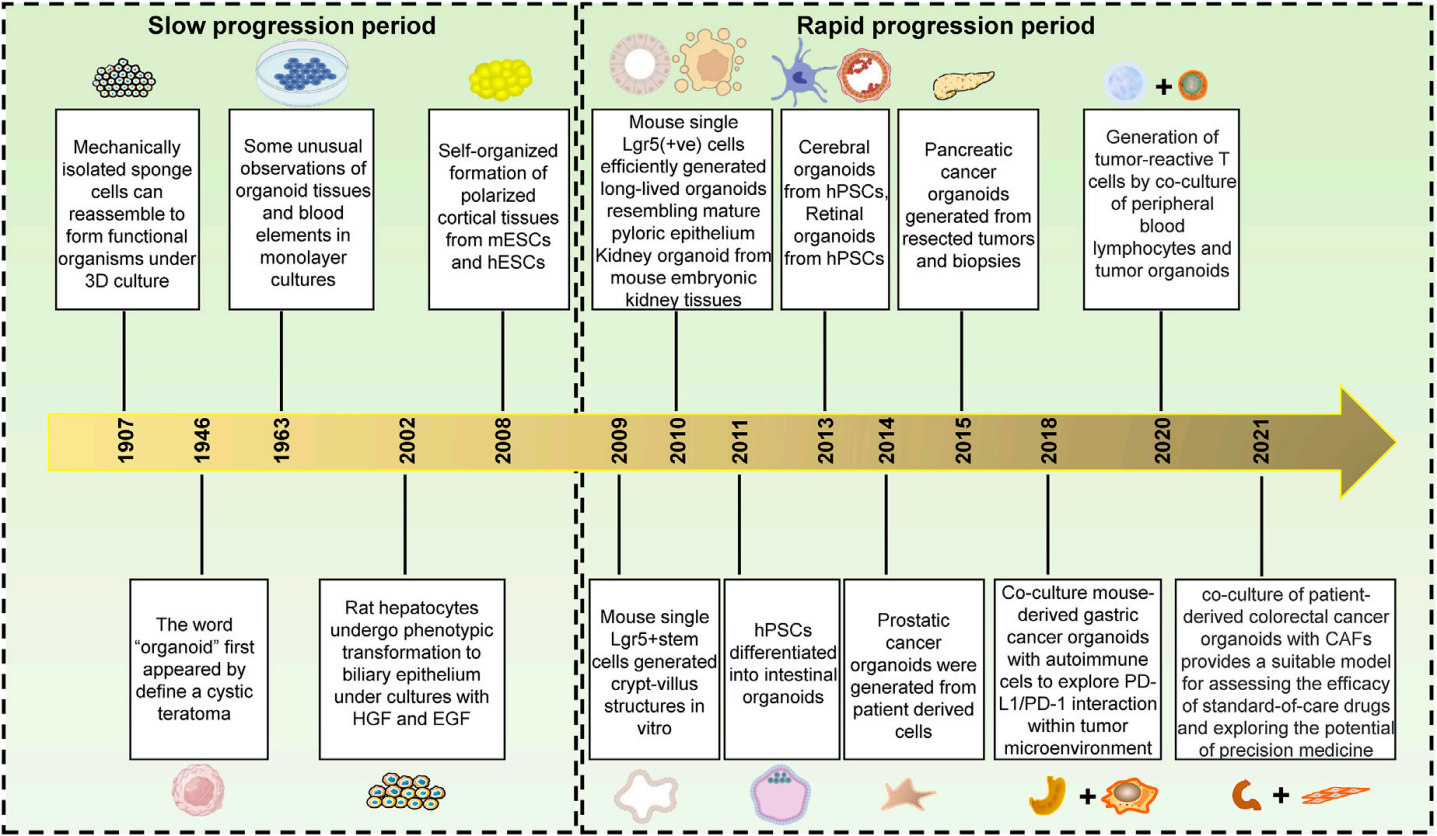
The history of organoids development. From 1907–2008, the development of organoids progressed slowly; however, from 2009 to present day, there has been a rapid advancement in organoid technology. This is evidenced by the initial successful culture of small intestinal crypt organoids and culminating in the maturation of tumor organoid co-culture. These milestones mark an increasingly widespread application of organoid technology within medicine.

Organoids were widely used in disease research in recent years, but the history of organoids development had undergone a long process of stagnation till the rapid development in the last decade (Figure 1). The origin of the organoids could be traced back to 1907, when H.V. Wilson, a professor at Baker Raleigh University, discovered mechanically separated sponge cells who could reassemble and self-organize into new functional sponge organisms under 3D culture (H.V.Wilson, 1907). This was the earliest report about organoid generation, however, the term “organoid” was first used to define cystic teratoma in a 2-month-old patient because it looked like an organ in appearance in 1946 (Smith and Cochrane, 1946). Subsequently, organoids were broadly defined as a class of cell clusters. In 1963, a blood vessel structure was observed in monolayers of cell cultures, suggesting that monolayers could also be cultured into different cellular compositions (Schneider et al., 1963). Nevertheless, the way in which organoids been induced was not well understood at that time. In 2002, researchers found that hepatocytes could spontaneously transform to biliary Epithelial Cells (BECs) under the condition of HGF and EGF addition (Michalopoulos et al., 2002). This discovery expanded the understanding of organoid induction and unlocked the key to organoid development rapidly. Additionally, in 2008, a study discovered that individual murine leucine-rich repeatcontaining G protein-coupled receptor 5 (LGR5+) intestinal stem cells could spontaneously form cortical tissue under certain conditions, and this process could be regulated by exogenous signals, this discovery laid the foundation for organoid development (Eiraku et al., 2008). Subsequently, a landmark study emerged in which researchers discovered that a single Lgr5+ stem cell could self-organize into an intestinal organ with intestinal crypt-villi structure through differentiation and self-assembly *in vitro*, suggesting that stem cells could self-organize and develop into functional organs under certain conditions (Sato et al., 2009). Since then, organoid researches entered a period of rapid development. In 2010, scientists found that Lgr5+ stem cells could be cultivated to form gastric organoids similar to pyloric epithelium *in vitro*, and noted that gastric organoids were important tools for studying gastric epithelial renewal, inflammation/disruption, and gastric cancer (Barker et al., 2010). In the same year, researchers discovered that single cell suspensions isolated from embryonic kidney tissues could transformed into a cell mass with the similar morphology and appropriate molecular markers to original tissues. Moreover, it connected distally to ureteral buds, marking the successful establishment of a kidney organoid (Unbekandt and Davies, 2010). One year later, human pluripotent stem cells were successfully grown to form intestinal organoids *in vitro*, and Wnt3A and FGF4 were discovered to be essential factors for intestinal organoids cultivation, which expanded the cell sources for organoids cultivation and accelerated the development of organoids (Spence et al., 2011). After that, retinal organoids developed from human pluripotent stem cells were successfully cultivation in 2013 (Bellapianta et al., 2022). In addition, it was also found that human pluripotent stem cells could spontaneously organize into brain organoids in matrigel (Lancaster et al., 2013). Since then, human prostate organoids, liver organoids, bile duct organoids, and lung organoids had been successfully cultivated (Chua et al., 2014; Tuveson and Clevers, 2019; Corro et al., 2020). Tumor organoids were also important direction for organoids development in recent year. Scientists successfully cultivated advanced prostate cancer organoids (Gao et al., 2014) and pancreatic cancer organoids (Boj et al., 2015) from biopsy tissue and circulating tumor cells. Thereafter, other tumor organoids such as glioblastomas organoids, colorectal cancer organoids, gastric cancer organoids and breast cancer organoids had been successfully cultivation (Hubert et al., 2016; Kretzschmar, 2021). Due to the absence of tumor microenvironment components such as immune cells, nerve cells and fibroblasts, tumor organoids are limited in their ability to mimic *in vivo* tumors. To explore the interplay between tumor organoids and their microenvironment, as well as the immune response of tumors, a co-culture system for tumor organoids was established (Yuan et al., 2022). Co-culturing mouse-derived gastric cancer organoids with autoimmune cells enables the investigation of PD-L1/PD-1 interactions within the tumor microenvironment (Chakrabarti et al., 2018). Additionally, researchers have co-cultured lymphocytes with tumor organoids to generate tumor-reactive T cells, which holds great potential for the immunotherapy of tumors (Dijkstra et al., 2018). Recently, a classical co-culture method of tumor organoids has been developed to predict the efficacy of precise treatment for gastric cancer patients, leading to an improved prognosis. This method utilizes tumor antigens to stimulate antigen-presenting dendritic cells (DCs), which are then co-cultured with CD8^+^ T cells in order to promote cytolysis and proliferation prior to co-culture with patient-derived gastric cancer organoids (Chakrabarti et al., 2021). Fibroblasts play a crucial role in the tumor-stromal interaction, co-culturing colorectal carcinoids with tumor-associated fibroblasts can enhance the plasticity of fibroblasts and provide insight into the role of tumor-associated fibroblasts in the progression of colorectal cancer (Luo et al., 2021). As the development of organoid technology, it is believed that more and more organoids will be successfully cultivated to help people better understand the pathogenesis of diseases and to provide ideal research models for disease treatment.

### The cultivation for tumor organoids

**FIGURE 2:**
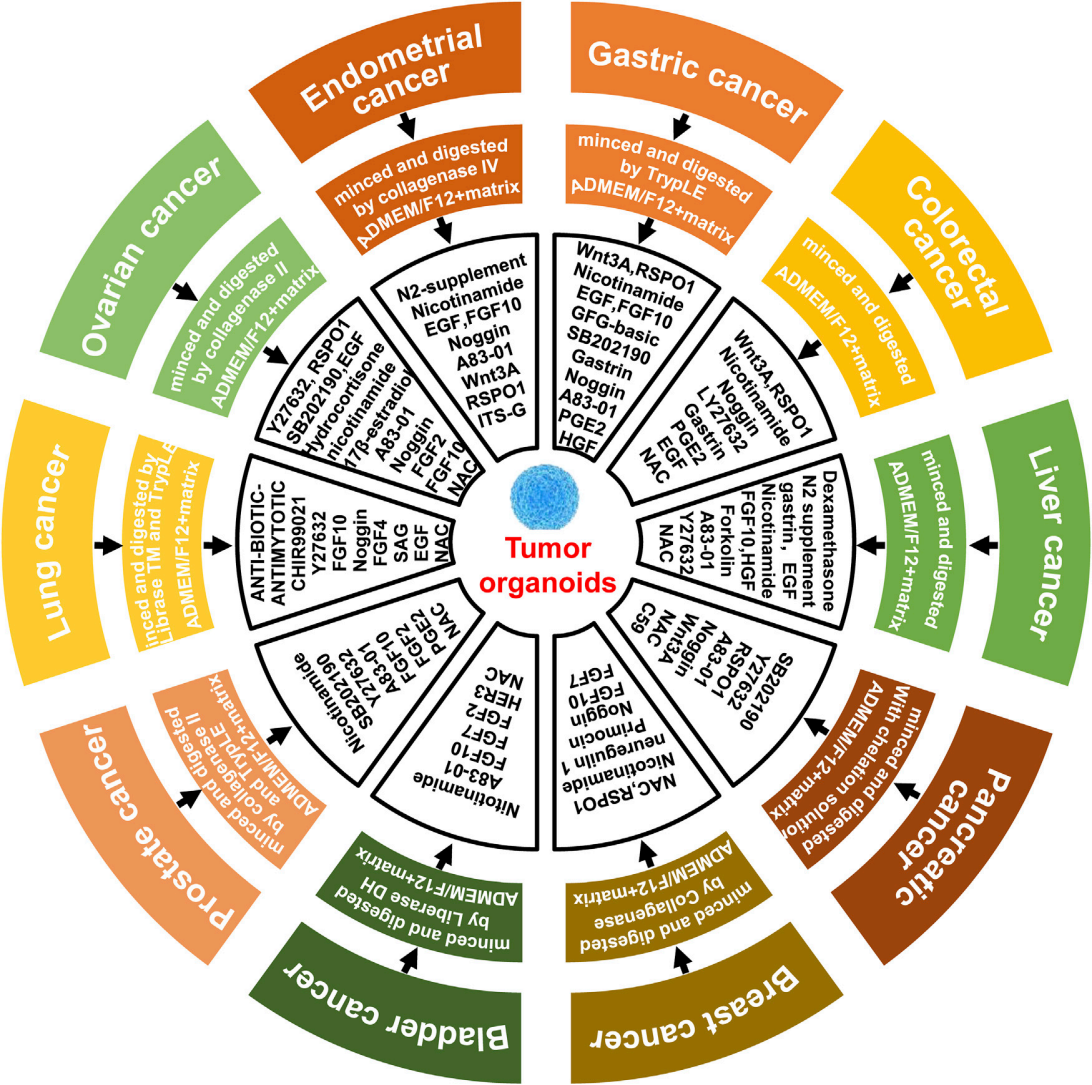
Summary of cytokines required for the cultivation of tumor organoids. In general, the establishment of organoid cultures involves the procurement of tumor tissue, followed by mechanical and enzymatic dissociation to obtain individual cells that are subsequently cultured in Advanced DMEM/F12 medium supplemented with basal membrane matrix and various cytokines. The specific cytokine requirements vary among different tumor types, including but not limited to gastric cancer (Vlachogiannis et al., 2018), colorectal cancer (van de Wetering et al., 2015), liver cancer (Broutier et al., 2017), pancreatic cancer (Seino et al., 2018), breast cancer (Sachs et al., 2018), bladder cancer (Whyard et al., 2020), prostate cancer (Zhou et al., 2021), lung cancer (Shi et al., 2020), ovarian cancer (Hill et al., 2018) and endometrial carcinoma (Boretto et al., 2019).

Tumors are important threat to human health and longevity, and it is also one of the hottest fields in medicine. Since the cultivation of small intestine organoids, organoid technology had been widely developed in biomedicine, and its great advantages been favored by tumor researchers. However, tumor organoids were difficult to cultivate, besides basal medium, numbers of cytokines were needed, furthermore, the cytokines needed for cultivating tumor organoids were varied between tumors (Figure 2) (Papapetrou, 2016).

Next, we will make a brief summary of the current methods for tumor organoids cultivation. Gastric cancer is the fifth most common malignancy among new cases of tumors worldwide, and its mortality ranks the 4th among deaths caused by tumors (Sung et al., 2021). Yet the cultivation of gastric cancer organoids was complicated. Besides using ADMEM/F12 (Advanced Dulbecco’s Modified Eagles Medium with Nutrient Mixture F12 Hams) and basement membrane matrix, B27, EGF, Noggin, R-Spondin 1, Gastrin, FGF-10, FGF-basic, Wnt3A, Prostaglandin E2 (PGE2), Y-27632 (Rock inhibitor), Nicotinamide, A83-01 (inhibitor of TGF-β type I receptor), SB202190 (p38 MAPK inhibitor) and HGF should also be added to ensure the successful cultivation of gastric cancer organoids (Vlachogiannis et al., 2018). As for the culture of colorectal cancer organoids, besides human Advanced DMEM/F12 and basic membrane matrix, it was necessary to add B27, N-acetyl Cysteine, R-Spondin1, Nicotinamide, EGF, Gastrin, A83-01, SB202190, Prostaglandine E2 and LY27632 (van de Wetering et al., 2015). It showed that the cytokines to be added for organoid cultivation of different tumors were verified. Primary hepatocellular carcinoma, one of the highest mortality rate malignant tumors, was mainly divided into hepatocellular carcinoma and cholangiocellular carcinoma (Llovet et al., 2021). For hepatocellular carcinoma, dexamethasone was used in the cell culture medium instead of the conventional Noggin, R-spondin-1 and Wnt3A. in addition, Forkslolin was necessary. While in cholangiocytic hepatocellular carcinoma, the addition of R-spondin-1 was required to maintain cell growth, which further reflected the complexity of the tumor organoids cultivation (Broutier et al., 2017). Unlike other tumor organoids, removal of EGF and adding of Wnt inhibitors could achieve better success rate in the culture of pancreatic cancer organoids (Seino et al., 2018). Breast cancer is the highest incidence tumor among women, with increasing new cases each year. The culture medium for breast cancer organoids was slightly different from other tumor organoids, such as Wnt3A, which was necessary for the culture of other tumor organoids, but dispensable for breast cancer organoids cultivation; while Neuregulin 1 was essential for maintaining efficient regeneration and long-term expansion of breast cancer organoids. EGF has double-sided effect on breast cancer organoids cultivation, low concentration of EGF inhibited cell proliferation but promoted tumor organoids self-organizing while high concentration of EGF promoted cell proliferation but caused tumor organoids disintegration and loss function of self-organizing. In addition, SB202190 was another important determinant for the establishment of breast cancer organoids (Sachs et al., 2018). Most bladder cancers were uroepithelial cancers and most of them were non-muscle invasive bladder cancers which usually had a good prognosis with high cost of treatment (Humphrey et al., 2016). The cultivation of organoids helped to reduce the cost of bladder cancer treatment. It was mainly done by using CTOS medium (containing advanced Dulbecco’s modified Eagle medium/F-12, B27, A83-01, N-acetylcysteine and nitotinamide). Moreover, it is necessary to supplement FGF10, FGF7, FGF2 and HER3 (Whyard et al., 2020). Prostate cancer is one of the most common types of cancer in men. According to the data in 2020, the risk of prostate cancer in men was 7.3%, only less than lung cancer; at the meantime, the death rate of prostate cancer reached to 3.8%, which became a major threat to men’s health (Siegel et al., 2021; Sung et al., 2021). For prostate cancer organoid cultivation, A83-01, FGF10, FGF2, prostaglandin E2(PGE2), Nicotinamide and p38 inhibitor SB202190, N-acetylcysteine, B27 and Rho kinase inhibitor Y-27632 were added into the general organoid culture medium. However, for mice prostate cancer organoids, FGF10, FGF2, PGE2, Nicotinamide and SB202190 were not necessary (Zhou et al., 2021). Lung cancer is the most common malignant solid tumor worldwide. Due to the importance and special nature of the lung organ in the human body, the clinical manifestations of lung cancer in the early stage were not obvious, which made it easier to confuse with other infections and thus overlooked. Usually, the tumor had already progressed to the middle and late stage when confirmed; therefore, timely and accurately screening of appropriate anti-tumor drugs was particularly important for lung cancer patients (Nasim et al., 2019). The cultivation of lung cancer organoids greatly reduced the time for drug screening. In addition to the common cytokines for lung cancer organoid cultivation, Antibiotic-Antimycotic, CHIR99021, FGF4, SAG (Smoothened Ligand) and Y27632 were needed (Shi et al., 2020). Similar to lung cancer, ovarian cancer is also a type of malignant tumor without obvious early symptoms, and about 70% of ovarian cancer patients had progressed to advanced stages when diagnosed, resulting in a 5-year survival rate of less than 40% (Yang et al., 2021). Currently, there were several methods to culture ovarian cancer organoids, but the highest success rate was achieved by using a combination of matrix gel and Advanced DMEM/F12 basal medium supplemented with Glutamax, B27, Nicotinamide, EGF, FGF2, FGF10, A83-01, N-acetylcysteine, Noggin, Rspondin1, p38 inhibitor, Hydrocortisone, and Y27632, resulting in 80%–90% success rate of carcinoid culture (Hill et al., 2018). Moreover, tumor organoids have been successfully cultivation for kidney cancer, endometrial cancer, cervical cancer and glioma (Boretto et al., 2017; Boretto et al., 2019; Grassi et al., 2019; Maru et al., 2019; Jacob et al., 2020).

As we can see from the different methods of tumor organoid establishment described above, the initial raw material for tumor organoids cultivation were tumor cells obtained by mechanical or enzymatic digestion from cancer tissues, and then special medium were used to culture these isolated tumor cells to generate different tumor organoids. The culture medium of these different tumor organoids almost all contained the same basal medium and cell growth factors, but only a few growth factors varied in different tumor organoids, indicating different preferences for organoid formation and reflecting the complexity among tumors (Aboulkheyr Es et al., 2018). And it also implied that these cytokines were the determinants of tumor organoids to be cultivated successfully. Conversely, these cytokines might also play the same function through artificial addition or replacement, so that the culture of different tumor organoids could be standardized, and then the purpose of large-scale batch production of tumor organoids can be achieved and widely used in clinical studies for tumors.

### Application of organoids in cancer

**FIGURE 3:**
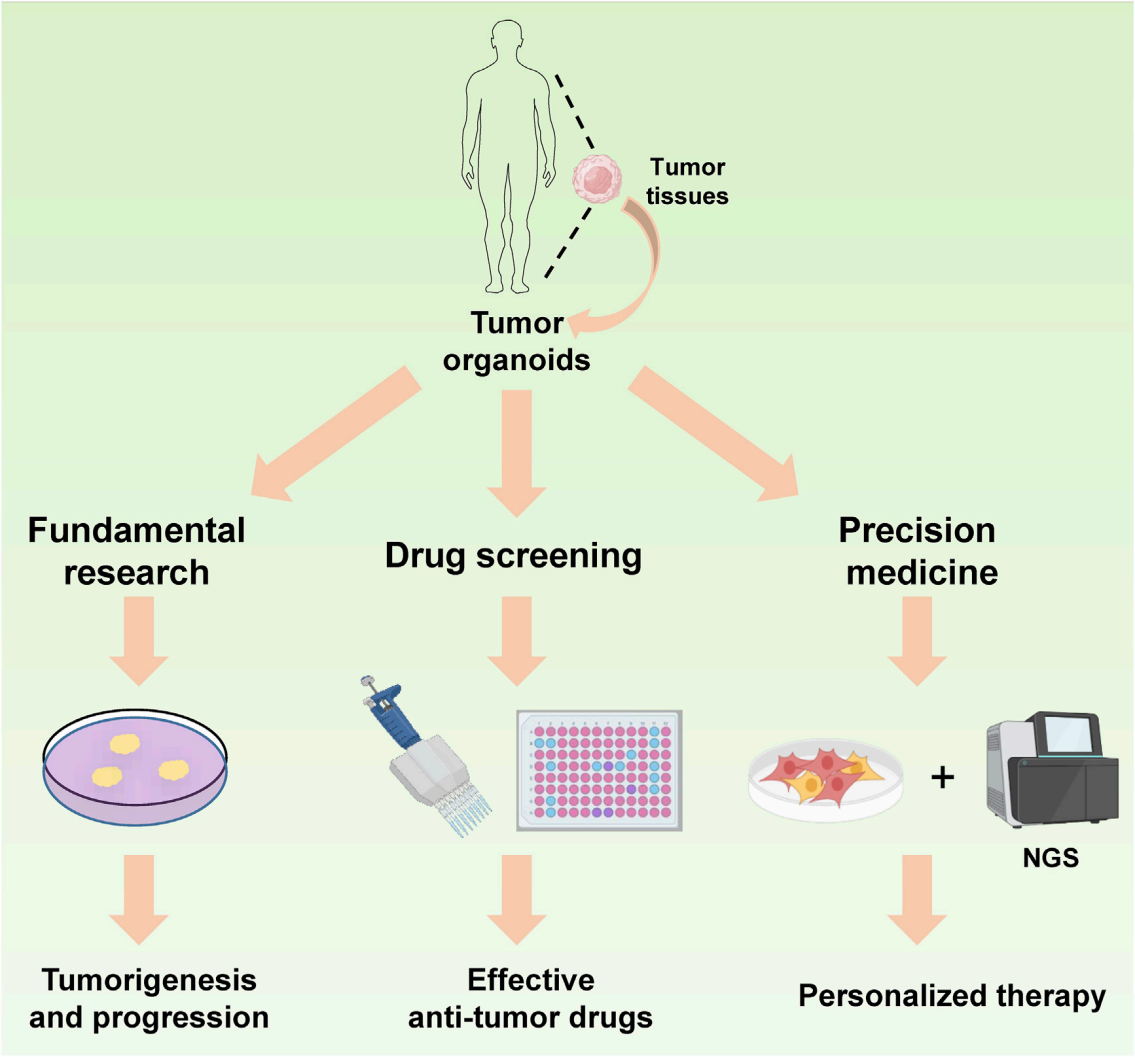
Application of organoids in cancer. The tumor organoids serve as an ideal model for investigating tumor progression. Moreover, owing to their inherited characteristics from parental tumor tissues and rapid culture ability, they can be effectively utilized for antitumor drug screening and play a crucial role in the precision treatment of cancer patients.

With the development and improvement of organoid technology, organoids were also widely used in the biomedical research especially in tumor research. The mechanism of tumorigenesis, the screening of anti-tumor drugs and the tumor precision therapy were three important directions and challenges in the field of oncology at present. Since their characteristic of spontaneously organization and preservation of genetic factors and tumor heterogeneity, tumor organoids were ideal tools for cancer research. In the following context, we reviewed the role of tumor organoids in tumorigenesis, screening of antitumor drugs, and tumor precision therapy (Figure 3).

### The role of organoids in the study of tumorigenesis mechanism

Tumor organoids can mimic the process of tumorigenesis, it implies that the mechanism of tumorigenesis could be studied through the cultivation of tumor organoids (Cimen Bozkus and Bhardwaj, 2021). Uhrf2 is high expressed in colorectal cancer, and its expression was positive associated with the grade and prognosis of colorectal cancer significantly (Lu et al., 2014). Uhrf2 deletion significantly reduced intestinal cancer organoid formation and reduced the number of tumor stem cells. Further study revealed that Uhrf2 could bind and SUMOylate TCF4, an important transcription factor downstream of the Wnt signaling pathway, thus stabilizing TCF4 expression and activating Wnt signaling, and finally Uhrf2 promoted the progression of intestinal cancer. Therefore, Uhrf2 was also considered as a target for the treatment of intestinal cancer (Muthusamy et al., 2020). Cancer-associated fibroblasts (CAF) are an important promoter in tumorigenesis, but the regulatory mechanism of tumor-associated fibroblasts is still unclear. Researchers found that tumor-secreted TGFβ and IL1 took an important role in the development of CAF heterogeneity via organoid model in pancreatic ductal adenocarcinoma. IL1 could produce inflammatory CAF by inducing LIF expression and activate downstream JAK/STAT signaling, while TGFβ antagonized this phenomenon by inhibiting IL-1R expression and promoting CAF differentiation into myofibroblasts (Biffi et al., 2019). In a bladder cancer organoid model, the used of the Wnt pathway activator CHIR99021 promoted bladder cancer cell proliferation and bladder cancer organoid formation significantly. In contrast, the use of RNAi to interfere the expression of β-catanin, an important protein in Wnt signaling, blocked bladder cancer organoid growth significantly, this confirmed the importance of Wnt signaling in bladder cancer growth (Yoshida et al., 2018). In an organoid model of intestinal cancer, researchers found that Wnt, Notch and Myb signaling took distinct roles in tumor development, it revealed pathway-dependent changes occurred during tumor initiation (Germann et al., 2014). By combining organoid technology and CRISPR/Cas9 technology, it is conductive to study the effect of gene mutations on tumorigenesis and development in a targeted manner. APC and TP53 genes are common mutated genes in intestinal cancer. When APC and TP53 are mutated in intestinal stem cells, a large number of aneuploidy cells can be found through the construction of organoid model, it was found that the organoids could develop into adenocarcinoma after tumor organoids transplanted into immunodeficient mice, this suggested that APC and TP53 gene mutations took an important driving role in the development of colorectal carcinogenesis. In contrast, transplantation of the same gene-edited organoid into the kidney capsule of mice resulted in tumor formation, albeit without metastasis to other organs, implying that the invasive behavior of tumors may necessitate involvement of additional genes (Matano et al., 2015). ARID1A mutations are among the most common gene aberrations in human cancers. The oncogenic consequences of ARID1A mutations in human cells are unclear due to the lack of forward genetic models. By combining gastric cancer organoids and CRISPR/Cas9 technologies, it was found that knockdown of ARID1A caused morphological dysplasia, tumorigenicity and mucus differentiation. In addition, the researchers identified BIRC5/survivin signaling as a key signal for early tumorigenesis caused by ARID1A knockdown (Lo et al., 2021). In CRC, researchers validated some CRC driver genes via utilizing CRISPR-Cas9 in instestinal tumor organoids and human CRC-derived organoids. They found that co-occurent mutations in receptors for activing and TGF-β synergistically promote tumorigenesis. Moreover, they found that Acvr1b, Acvr2a and Arid2 could function as tumor suppressor genes in CRC (Takeda et al., 2019). Another study had also showed that CRISPR-Cas9 technology could applied in patient-specific functional genomics screening, they screened and identified TGFBR2 as the most revalent tumor suppressor gene (Michels et al., 2020). It is indicated that the combination of organoid and gene editing technology could better investigate the role of tumor driver genes in the development of tumorigenesis.

### The role of organoids in the screening of anti-tumor drugs

Drug screening is an important part of disease treatment, and it will spend lot of time and money to screen for an effective clinical drug, which mainly due to the extremely low success rate (Kondo and Inoue, 2019). A study analyzed 406,038 clinical trials and 21,143 compounds involved in this clinical trials between 2000 and 2015 and found that the overall clinical trial success rate was only 13.8%, while the success rate for antitumor drug screening was even lower, it was only 3.4% (Wong et al., 2019). Drug screening consists of three parts: the candidate compounds or drugs, the screening methods, and the drug screening model, and the drug screening model is the most critical part among them, which is the object of drug treatment (Pushpakom et al., 2019; Tyers and Wright, 2019). Since the first report on the use of leukemia animal model for drug screening in 1950, an increasing number of human tumors had been transplanted into mice to construct mouse models for the screening of antitumor chemotherapeutic drugs (Yoshida, 2020). The most common model was the transplantation of a tumor cell lines into a mouse subcutaneously. However, these models could not truly mimic the growth of tumors due to the single cellular component of tumor cell lines and the dominant growth of tumor cells after passing through generations, which were mostly used for the study of tumor pathogenesis (Hutchinson and Kirk, 2011). In addition, *in situ* tumor transplantation model had also been developed for antitumor drugs screening. The patient-derived tumor tissues were digested or simply cut into small pieces and inoculated into the subcutaneous of mouse, known as the PDX (patient-derived xenograft) model. But it had the deficiency of time-consuming and difficulty in constructing, moreover, the cost was huge to construct PDX model (Hidalgo et al., 2014). Similarly, traditional 2D culture model had deficiency of single composition and susceptibility during long-term culturing. They were not the ideal models for antitumor drugs screening (Pereira and Williams, 2007; Calvert et al., 2016).

In recent years, with the rapid development of tumor organoid technology, a new anti-tumor drug screening model had been established, it was called tumor organoid model. The tumor organoid model had the characteristics of short time consuming, inexpensive and better mimicked the growth of tumor. It could be used as an effective biological model for preclinical studies and is widely appreciated. A study on a non-small cell lung cancer organoid model found that organoids constructed from surgically resected primary non-small cell lung cancer tissues preserved the tumorigenic properties of the original tumor tissues, such as the cytological characteristics of the malignancy and xenograft formation. In addition, whole-exome sequencing and RNA sequencing also showed that the organoids preserved the mutations and copy number aberrations from the parental tumor tissues, demonstrating the consistency in tumor cell morphology and genetic specificity between organoids and parental tumor tissues. Furthermore, organoid model retains the same sensitivity to tumor-targeted therapy as the parental tumor tissues, which demonstrates the reliability of the tumor organoid model in anti-tumor drug screening (Shi et al., 2020). Another study on lung cancer organoids showed that a large number of lung cancer organoids could be generated simultaneously by combining organoid technology and integrated superhydrophobic microwell array chip (InSMAR-Chip) technology. It was sufficient to produce a clinically meaningful drug response within a week. The drug test result fitted well with xenografts from cancer tissues in tumor gene mutations and drug sensitivity, which also provided a viable assay for anti-tumor drug screening and an important direction for anti-tumor drug screening (Hu et al., 2021). The establishment of tumor organoid biobank had greatly facilitated the screening of antitumor drugs (Table 1). In colorectal cancer, 83 compounds had been screened simultaneously by constructing a colorectal cancer organoid biospecimen library. Cetuximab was found to be highly efficacious for KRAS wild-type organoids, while Nutlin-3a was also highly effective in killing TP53 wild-type organoids, it was consistent with the results in clinical trials (van de Wetering et al., 2015). In gastric cancer, 37 compounds were examined by constructing a library of patient-derived gastric cancer organoid biospecimens, Some new potent compounds such as Abemaciclib, Napabucasin and ATR inhibitor VE-822 had been identified and showed antitumor effects in clinical trials (Yan et al., 2018). In hepatocellular carcinoma, 129 classes of antitumor drugs were tested by constructing a library of liver cancer organoid samples. In addition to FDA-approved drugs for liver cancer treatment, liver cancer organoids were found to be significantly sensitive to some new anti-tumor drugs (for which liver cancer was not indicated) (Li et al., 2019). In breast cancer, some signaling pathway inhibitors were detected by combining breast cancer organoids and gene sequencing technology, it was found that the sensitivity of breast cancer organoids to drugs was consistent with *in vivo* models of transplanted tumors in breast cancer patients as well as patient-derived mice (Sachs et al., 2018). A screening of 45 compounds for breast cancer found that docetaxel got an excellent result (Guillen et al., 2022). Moreover, a satisfactory result was obtained by testing the sensitivity of tumor organoids to drugs in bladder cancer (Lee et al., 2018) and ovarian cancer (Jabs et al., 2017). In a pancreatic cancer organoid drug screening study, researchers screened 76 non-clinical compounds and found that PRMT5 inhibitor EZP015556 could be a potential candidate for MTAP-negative pancreatic cancer (Driehuis et al., 2019). In addition, the enormous potential of tumor organoids for oncological drug screening has been demonstrated in prostate cancer and endometrial cancer, either alone or in combination with other drugs (Kiyohara et al., 2016; Karkampouna et al., 2021). Genetic mutations are a frequent feature of colorectal cancer. A study of colorectal carcinoids with KRAS mutations showed that the combination of KRAS inhibitors and EGFR-MEK-ERK pathway inhibitors could significantly inhibited organoids growth, which was also validated in a normal colorectal organoid model with KRAS mutations introduced by CRISPR technology (Verissimo et al., 2016). In tumors, various mutations can also lead to variations in the sensitivity of tumors to drugs. In a test of drug sensitivity in lung cancer patients, the researchers screened 26 antitumor drugs using lung cancer organoids and found that organoids with mutations in EGFR responded to gefitinib and osimertinib differently (Chen J H et al., 2020).

**TABLE 1:** The application of organoids in anti-tumor drug screening.

Tumor type	Model	Numbers of compounds	Excellent compounds	References
Colorectal cancer	Colorectal cancer organoids	83	Nutlin-3a	van de Wetering et al. (2015)
Gastric cancer	Patient-derived gastric cancer organoids	37	Abemaciclib Napabucasin VE-822	Yan et al. (2018)
Hepatocellular carcinoma	Liver cancer organoids	129	bortezomib	Li et al. (2019)
Breast cancer	Breast cancer organoids	45	Docetaxel	Guillen et al. (2022)
Bladder cancer	Blader cancer organoids	50	Trametinib SCH772984	Lee et al. (2018)
Lung cancer	Lung cancer organoids	26	Gefitinib Osimertinib	Chen Y et al. (2021)
Pancreatic cancer	Pancreatic cancer organoid	76	EZP015556	Driehuis et al. (2019)
Ovarian cancer	Ovaran cancer organoids	22	NA	Jabs et al. (2017)
Endometrial cancer	Endometrial cancer organoids	79	YM155	Kiyohara et al. (2016)
Prostate cancer	Prostate cancer organoids and PDX	74	ponatinib	Karkampouna et al. (2021)

These all indicated the reliability of tumor organoids in anti-tumor drug screening. In particular, the combination of organoid technology with high-throughput technologies such as chip technology would greatly expand the number of tumor samples while preserving tumor heterogeneity. It was beneficial for large-scale drug screening and improved the efficiency and scope of drug screening, organoids are important tool for future antitumor drug development and clinical application.

### The role of organoids in tumor precision therapy

With the development of biomedicine, it was found that drug response of tumor patients varied even though drugs were treated at the same dose, especially for some target-specific antitumor drugs (Ward et al., 2021). Further studies revealed that there are various mutations in the tumor tissues of different tumor patients, which might be the reason why anti-tumor targeted drugs did not work well for partial patients (Chen and Chen, 2020). The precision therapy of tumors is an effective response to the current clinical treatment of tumors. However, because of the complexity and heterogeneity of tumors, detection of mutations in a single target alone did not truly reflect the complexity of mutations in tumors. Moreover, new mutations were producing when treated with targeted anti-tumor drugs because of tumor adaptability, which were harmful for tumor patients (Xu et al., 2022). The emergence of tumor organoid model can greatly alleviate this situation. Patient-derived xenografts (PDX) model is the most ideal preclinical diagnostic model for tumors, However, due to the lengthy and costly of PDX model and its low success rate, it is not the best choice. Moreover, PDX requires more tumor tissue as the material for construct a PDX model.

While tumor organoids are much shorter and less expensive to construct compared to PDX model, and the success rate of construction is much higher than that of PDX model (Shiihara and Furukawa, 2022). Moreover, the construction of tumor organoids model demands few tumors tissue samples, even only tumor puncture samples (needle biopsy) can meet the requirements. At the same time, tumor organoids can also greatly retain the characteristics of the parental tumor, a variety of anti-tumor drugs can be screened at the same time using tumor organoids model, so that the most suitable anti-tumor drugs for patients can be screened in a short time, which can greatly improve the therapeutic effect of tumor patients and prolong the life cycle of patients (Ren et al., 2022). In a study for prostate cancer organoid, targeting organoid model of prostate cancer, researchers had found that multiple FDA-approved drugs, including multi-kinase inhibitors (ponatinib, sunitinib, sorafenib), could be effectively tested to screen for the most appropriate therapeutic agent by constructing organoid model (Karkampouna et al., 2021). In another study of organoid biospecimen banking in lung cancer patients, researchers have found that the most suitable anti-tumor drugs could be selected by screening tumor organoids, because tumor organoids retain the genetic characteristics of parental tumor tissues. For example, for BRCA2-mutated organoids, the use of olaparib could achieve better results, while for EGFR-mutated organoids, erlotinib was better. When EGFR mutations and MET amplification exist at the same time, crizotinib was the best choice (Kim et al., 2019). In a study of colorectal cancer organoid, the researchers tested 85 compounds (including targeted therapy compounds) in intestinal tumor organoids to explore the sensitivity of tumor organoids to chemotherapy drugs. It was found that TP53-deficient colorectal organoids were highly resistant to MDM2 inhibitors, whereas RAS-mutated colorectal organoids were insensitive to EGFR inhibitors. Furthermore, RNF43-mutated colorectal organoids were particularly sensitive to inhibitors of Wnt signaling, suggesting a classical approach to RNF43-mutated colorectal cancer (Aboulkheyr Es et al., 2018). Additionally, organoid had enabled the screening of antitumor drugs with fewer side effects, thus effectively reduced the side effects during antitumor treatment. In an organoid culture for colorectal diseases, researchers had cultured colonic organoids from induced pluripotent stem cells from patients with familial adenomatous polyposis (FAP-iPSCs). Antibiotic geneticin was screened out by this drug screening platform, it can effectively target colorectal cancer organoids with abnormal Wnt activity, but does not affect the growth of normal APC-mutated colonic organoids. It exhibited the important role of tumor organoids in drug selection for colorectal diseases (Crespo et al., 2017). Tumor organoids had also shown their advantage in renal cell carcinoma. Multiple anti-tumor drugs such as sunitinib, pazopanib, cabozantinib, axitinib and sorafenib could be screened for tumor treatment by constructing patient-derived renal cell carcinoids (Kazama et al., 2021). Malignant rhabdomyosarcoma (MRT) is one of the most aggressive childhood malignancies, but there are currently no effective treatment options for malignant rhabdomyosarcoma and the prognosis is poor. By constructing an organoid model of malignant rhabdomyosarcoma and using the organoid model of normal tissue as a control, it could be used to screen for antitumor drugs that effectively inhibit malignant rhabdomyosarcoma. A neddylation inhibitor MLN4924 was screened out by this model and proved that a single dose of MLN4924 could significantly prolong survival of patients. Exhibiting the advantages of tumor organoid in screening antitumor drugs with few sides’ effects (Calandrini et al., 2021). In ovarian and breast cancers, organoids also offered advantage in screening antitumor drugs (Kopper et al., 2019; Chen P et al., 2021).

Apart from *in situ* tumors, tumor organoids can also be used for drug sensitivity analysis by culturing tumor cells in malignant effusions to form tumor organoid *in vitro*. An experimental study on malignant effusion specimens from patients with high-grade serous ovarian cancer showed that by recovering multicellular spheroids in malignant effusion and culturing organoids, effective therapeutic drugs could be screened for individualized treatment of patients via this organoids (Chen H et al., 2020). The association of genes with drugs took a key role in individualized and targeted tumor therapy. A strong correlation between gene mutations and the therapeutic effect of chemotherapeutic drugs had been demonstrated and referred to mutation-based drug sensitivity (MDS). Because genomic analysis was not sufficient to identify effective treatment options for most patients with advanced cancer, drug screening of tumor organoids can clarify unknown drug responses (Pauli et al., 2017).

### Disadvantages of organoids in cancer therapy

Tumor organoids have the advantages including short culture time, low cost, and high construction success rate; however, they also have some deficiencies. For example, Although the overall success rate of tumor organoids construction was higher compared to the PDX tumor model, but they were uneven and depended on the type of tumors. In addition, it was also highly susceptible to contamination during the cultivation of tumor organoids (Dijkstra et al., 2020). Until now, there has been a lack of uniform standards for the cultivation of organoids, slight differences in organoid culture methods and culture conditions established by each laboratory result in diverse success rates. Furthermore, tumor organoids were built up in a shorter period of time compared to PDX model, it still took a short period of time for organoid model construction. When the tumor organoids were used for screening appropriate drugs or other analysis for patients those with mid to late-stage tumors, there was not much time to wait for the best drug match results (Veninga and Voest, 2021). Moreover, the cultivation of organoids requires special medium as well as the addition of different cytokines and inhibitors, which were also costly. In addition, among these inhibitors required for tumor organoids, some of them were also signaling pathway inhibitors, such as ALK inhibitor A83-01 and P38 inhibitor SB202190. And these inhibitors could be adverse factors for drug screening against related signaling pathways (Grassi et al., 2019; Veninga and Voest, 2021). Another problem was the lack of fibroblasts, immune cells and nerves in the tumor organoid model, they played an important role in tumor growth (Neal et al., 2018; Cattaneo et al., 2020; Kim et al., 2020). For example, tumor-associated fibroblasts could secrete cytokines and promote tumor growth and abnormal tumor-associated stroma (Chen Y et al., 2021), Immune cells, such as M2 macrophages, are also critical to the malignant progression of tumors (Entezari et al., 2022). Therefore, the organoid model could not display the tumor microenvironment completely and reality, it was more outstanding when immunotherapy drugs were screened (Xia et al., 2019). Matrix is an important support of most tumor organoids, most of them were cultured using animal-derived mechanisms or collagen, but these matrices contain many unknown growth factors in addition to laminin, collagenIV and entactin. These were the safety hazards in clinical applications (Gjorevski et al., 2016; Blondel and Lutolf, 2019).

## Conclusion

Tumor organoid model was widely used in recent years, they were produced by using stem cell-derived tissues (embryonic stem cells, induced pluripotent stem cells, and tumor stem cells) and cultivated under specific conditions (Garreta et al., 2021). Organoids met the needs of biomedical research in many aspects, including disease development mechanism, drug screening, personalized therapy and tissue regeneration (Artegiani and Clevers, 2018). This paper reviewed the history of organoid, the methods for organoids establishment, the application of organoid in oncology (including tumor organoid in basic tumor research, the role of tumor organoid in drug screening and the role of organoid in tumor precision therapy) and discussed the shortcomings of current organoid in oncology research. We found that tumor organoids played an important role in exploring the occurrence and development of tumors. Whole genome sequencing and RNA sequencing have demonstrated that the organoid maintains the genetic characteristics of the original tumor tissue, including its genetic mutations and copy number variations. Additionally, the organoid exhibits histopathological features consistent with those of the parental tumor tissue (Romero-Calvo et al., 2019; Shi et al., 2020). They were the ideal model for tumor research as they could reflect the occurrence and development of tumors more realistically. However, there were few clinical trials were operated via tumor organoids, which might be related to the complicated and requirements of tumor organoid establishment. It is believed that the potential of tumor organoids in basic tumor research would be further explored with the development of organoid technology. As we all know, the screening of antitumor drugs was a long and costly process and conventional 2D cultured cells were widely used with less effective, resulting in a great waste of human and financial resources. Because long-term culturing would lose some genetic information and produce new mutations in tumor cells. Moreover, limited by the 2D culture dish, the 3D environment of tumor growth cannot be truly reflected as the target cells were relatively single, which cannot truly simulate the tumor progression. Therefore, most of the anti-tumor drugs were effective in 2D culture model but not in clinical trials (Ben-David et al., 2017; Byrne et al., 2017; Zhou et al., 2017). In contrast, tumor organoids had more advantage in drug screening because they cultured in a 3D growth environment that preserved the heterogeneity and growth characteristics of the origin tumor tissues, they could mimic the environment of tumor reality. Meanwhile, for tumor patients, especially those whose tumors had progressed to the middle or late stages, the tumor organoids could screen out the appropriate anti-tumor drugs quickly. Due to the ability of organoids to simultaneously screen a large number of antitumor drugs and their short screening period, they are capable of efficiently identifying the most suitable chemotherapy drugs for patients in a timely manner. Therefore, they are highly applicable for precision therapy in tumor treatment. However, we also found an important problem with the current tumor organoids technology-the success rate of building tumor organoids varied greatly among different tumor types and different subject groups (Drost and Clevers, 2018). The reason was that there was no uniform standard for the establishment of tumor organoids currently. The cytokines used for tumor organoid culture were varied among tumors and research groups, and it was difficult to construct tumor organoids on a large scale successfully, at the same time, it would limit the application of tumor organoids. Furthermore, the lack of tumor-associated fibroblasts, immune cells, nerve cells and blood vessels in tumor organoids could not fully mimic the tumor microenvironment, which was another important factor to restrict tumor organoids application (Workman et al., 2017; LeSavage et al., 2022). Fortunately, researchers were currently trying to solve this problem by co-culturing organoid cells with immune cells and fibroblasts, and had achieved good outcomes in tumor organoids (Cattaneo et al., 2020; Zhao et al., 2021; Sun et al., 2022). Overall, we summarized the role of organoid technology in tumor development, drug screening and precision therapy. Although there are still many problems of tumor organoids, it is believed that these problems will be overcome with the research progresses, and tumor organoids might be a powerful tool for tumor treatment.

## Abbreviations

2D, 2 dimensional; BECs, biliary Epithelial Cells; CAF, Cancer-associated fibroblasts; FAP, familial adenomatous polyposis; InSMAR-Chip, integrated superhydrophobic microwell array chip; LGR5, leucine-rich repeatcontaining G protein-coupled receptor 5; MDS, mutation-based drug sensitivity; MRT, Malignant rhabdomyosarcoma; PDX, patient derived xenograft; PGE2, prostaglandin E2.
